# P-2092. Evaluation of Daptomycin for MicroScan Dried Gram Positive MIC Panels from a Multicenter Assessment of Select Gram Positive Species

**DOI:** 10.1093/ofid/ofae631.2248

**Published:** 2025-01-29

**Authors:** Kristianne Dawa, Regina Brookman, Jose Diaz, Christine Hastey

**Affiliations:** Beckman Coulter, West Sacramento, California; Beckman Coulter, Inc., West Sacramento, CA, California; Beckman Coulter, Inc., West Sacramento, CA, California; Beckman Coulter, West Sacramento, California

## Abstract

**Background:**

Given the need for accurate antimicrobial susceptibility testing results to support patient care decisions, data were evaluated from clinical isolates of *Staphylococcus aureus* and *Enterococcus faecalis* on an investigational MicroScan Dried Gram Positive MIC (MSDGP) panel with daptomycin.*

**Figure:**
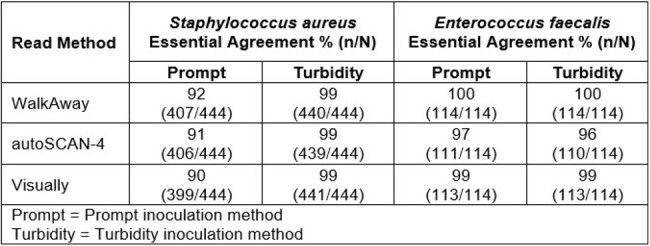

**Methods:**

Data from a previous multicenter study conducted at four sites were analyzed by comparing MIC values obtained using the MSDGP panels to MICs from a CLSI broth microdilution reference panel. A total of 444 *Staphylococcus aureus* and 114 *Enterococcus faecalis* clinical isolates were analyzed. These isolates were tested using the turbidity and Prompt methods of inoculation on MSDGP panels. The panels were incubated at 35 ± 1^°^C and read on the WalkAway System, the autoSCAN-4 instrument, and visually. Read times for the MSDGP panels were at 16-20 hours. Frozen reference panels were prepared and read according to CLSI methodology.

**Results:**

Essential agreements were calculated comparing MIC results from the MSDGP panels with MIC results from frozen panels for all isolates tested in efficacy and challenge phases and are found in the following table.

**Conclusion:**

Daptomycin MIC results for *Staphylococcus aureus* and *Enterococcus faecalis* obtained with the MSDGP panel correlate well with MICs obtained using frozen reference panels in this multicenter study.

*Pending submission and clearance by the United States Food and Drug Administration; not yet available for in vitro diagnostic use in the US.

For Investigational Use Only. The performance characteristics of this product have not been established.

©2024 Beckman Coulter. All rights reserved. All other trademarks are the property of their respective owners.

Beckman Coulter, the stylized logo, and the Beckman Coulter product and service marks mentioned herein are trademarks or registered trademarks of Beckman Coulter, Inc. in the United States and other countries.

May be covered by one or more patent. See www.beckmancoulter.com/patents.

2024-12909

**Disclosures:**

Kristianne Dawa, n/a, Beckman Coulter: employee Regina Brookman, BS, Beckman Coulter: employee Jose Diaz, BS, Beckman Coulter: employee Christine Hastey, PhD, Beckman Coulter: employee

